# Corneal Epithelial Thickness Mapping: A Major Review

**DOI:** 10.1155/2024/6674747

**Published:** 2024-01-02

**Authors:** Mohammad-Ali Abtahi, Amir Hushang Beheshtnejad, Golshan Latifi, Marjan Akbari-Kamrani, Sadegh Ghafarian, Ahmad Masoomi, Seyed Ali Sonbolastan, Hamidreaza Jahanbani-Ardakani, Mehrnaz Atighechian, Laleh Banan, Hosein Nouri, Seyed-Hossein Abtahi

**Affiliations:** ^1^Farabi Eye Hospital, Tehran University of Medical Sciences, Tehran, Iran; ^2^Department of Ophthalmology, School of Medicine, Shiraz University of Medical Sciences, Shiraz, Iran; ^3^Sunshine Coast University Hospital, Brisbane, Queensland, Australia; ^4^Ophthalmic Research Center, Research Institute for Ophthalmology and Vision Science, Shahid Beheshti University of Medical Sciences, Tehran, Iran

## Abstract

The corneal epithelium (CE) is the outermost layer of the cornea with constant turnover, relative stability, remarkable plasticity, and compensatory properties to mask alterations in the underlying stroma. The advent of quantitative imaging modalities capable of producing epithelial thickness mapping (ETM) has made it possible to characterize better the different patterns of epithelial remodeling. In this comprehensive synthesis, we reviewed all available data on ETM with different methods, including very high-frequency ultrasound (VHF-US) and spectral-domain optical coherence tomography (SD-OCT) in normal individuals, corneal or systemic diseases, and corneal surgical scenarios. We excluded OCT studies that manually measured the corneal epithelial thickness (CET) (e.g., by digital calipers) or the CE (e.g., by confocal scanning or handheld pachymeters). A comparison of different CET measuring technologies and devices capable of producing thickness maps is provided. Normative data on CET and the possible effects of gender, aging, diurnal changes, refraction, and intraocular pressure are discussed. We also reviewed ETM data in several corneal disorders, including keratoconus, corneal dystrophies, recurrent epithelial erosion, herpes keratitis, keratoplasty, bullous keratopathy, carcinoma in situ, pterygium, and limbal stem cell deficiency. The available data on the potential role of ETM in indicating refractive surgeries, planning the procedure, and assessing postoperative changes are reviewed. Alterations in ETM in systemic and ocular conditions such as eyelid abnormalities and dry eye disease and the effects of contact lenses, topical medications, and cataract surgery on the ETM profile are discussed.

## 1. Foreword

The corneal epithelium (CE) is the outermost layer of the cornea, with constant turnover and relatively stable thickness in normal eyes. In continuous relation to the precorneal tear film, this layer provides protective and optical properties vital to maintaining a healthy ocular surface [1]. In contrast to this relative stability in normal eyes, epithelium alters to mask the changes of the underlying stroma with great plasticity [2, 3]. In this regard, recognizing the healing and remodeling patterns of the corneal epithelium after refractive surgery may illuminate the exact cause of residual refractive errors after different types of laser refractive surgery (LRS) [3–5] or orthokeratology (OK) [6]. Furthermore, the compensatory role of the CE in masking the underlying irregularities has been proven in corneal ectasia and can help recognize subtle ectatic changes [7, 8]. Based on the above and the advent of accurate diagnostic tools, distinguishing different patterns of epithelial remodeling has become a topic of interest in the last decade.

Measurement of CE thickness (CET) in the current literature can be categorized into two main methods: (1) CET point measurement and (2) CET map, i.e., epithelial thickness map (ETM).

Devices that can produce repeatable ETMs should be able to acquire the image rapidly to minimize motion artifacts and analyze them in larger areas. Although confocal microscopy has an excellent axial resolution, it cannot have a holistic view of the cornea to produce maps [9]. The same is true about handheld ultrasound pachymeters [10]. On the other hand, by gathering and computing data from a wider area of the cornea, very high-frequency digital ultrasound (VHF-US) technology and optical coherence tomography (OCT) can produce maps from a larger diameter of the cornea [11].

Pioneering in the introduction of ETM, Reinstein et al. introduced and promoted a unique VHF-US device to provide a sophisticated view of the corneal epithelium [1, 3, 4, 11–36]. They first presented a prototype of their device in 1994, mapping the 3 mm of the central cornea; by promoting it in 1998, the mapping area became wider to a diameter of 10 mm [11, 33]. The device was approved by US Food and Drug Administration with the brand name Artemis VHF-US (ArcScan Inc., Morrison, CO). It employs a 50 MHz probe to make a precise measurement of the CE with high precision (measurement repeatability ∼ 0.58  *μ*m) [24]. In the modern device setting, the patient is in the sitting position while the eye is immersed in 33°C balanced salt solution (BSS) in a soft eye cup without using an eyelid speculum. To measure the epithelium with VHF-US, the required CET should be at least 21 *μ*m; the precision of the device is approximately 1 *μ*m [11].

The early time-domain- (TD-) OCT machines lacked enough speed to produce a map of the CET and could be used to evaluate the CET point measurement like handheld pachymeters [37–39]. Hence, the distance between the first and second spikes was considered corneal epithelium. Haque and associates [40] were the first to produce ETM using a TD-OCT device. With the introduction of fast,high-resolution devices, including Fourier-domain (FD) and spectral-domain- (SD-) OCT, along with automated image processing [41, 42], the automatic mapping of the corneal layers, including CE, became feasible. In 2012, RTVue SD-OCT (Optovue, Inc., Fremont, CA) was introduced as the first commercially available OCT device capable of mapping the CE [7]. The resolution of this device is about 5 *μ*m, three times better than TD-OCT but still lower than VHF-US technology [8, 11].

This comprehensive synthesis aimed to gather and review all possible data—of various evidence levels—on epithelial thickness mapping with different imaging methods in normal individuals and in corneal/systemic diseases. We reviewed the reproducible ETM studies provided by VHF-US and OCT technologies. We excluded the OCT studies that used corneal cross sections to measure CET manually by digital calipers or through evaluating the CE with confocal scanning or handheld pachymeters.

## 2. CET Measurement Technologies: Different Devices and Repeatability

### 2.1. OCT vs. VHF-US Technology

The first important difference between these two technologies is the noncontact nature of the OCT device as opposed to the need to use immersion in VHF-US; it has the advantage of no chance of infection transmission and no discomfort for the patient. The second difference is the longer acquisition time in VHF-US, which is about two to three minutes compared to less than one second in SD-OCT technology (Table 1). The other difference between these two technologies is the incorporation of the tear lake with the immersion fluid in VHF-US, which omits the measurement of the tear layer in this technology. Reinstein et al. compared the ETM data produced by Artemis VHF-US and RTVue SD-OCT devices in 189 virgin eyes and 175 post-laser refractive surgery (LRS) eyes. Although CET measurements in OCT include tear film thickness, they found thinner measures by SD-OCT technology in 70% of virgin and 85% of post-LRS corneas. They found the average CET in virgin and post-LRS eyes to be 0.71 *μ*m and 2.48 *μ*m thinner by OCT, respectively. Taking the tear film thickness into account, they concluded that mean CET measurements are about 4 to 6 *μ*m thinner in OCT. The agreement of the measurements between the two devices was fairly close [11]. Another similar study by the same group had the same results in normal eyes [51]. The higher mean CET value in VHF-US was postulated as a result of swollen corneal tissue immersed in BSS, which authors ruled out in another study [19].

Other differences between the two devices include higher axial resolution of VHF-US (about 1 *μ*m vs. 3.6–5 *μ*m in SD-OCT devices) [8, 43] and larger diameter of evaluation (10 mm vs. 6–9 mm in different OCT devices) [43, 52].

### 2.2. OCT vs. Confocal Microscopy

Although confocal devices are unable to produce ETM with current technology, a study compared point measurements done by this technology with ETM produced by SD-OCT and found significantly higher average CET measurement using SD-OCT (55.6 ± 4.0 *μ*m) compared to confocal microscopy (51.9 ± 4.9 *μ*m). The authors attributed the difference to OCT devices taking the tear film into account [9].

### 2.3. Different Devices Capable of Producing ETM

In 2012, RTVue SD-OCT (Optovue, Inc., Fremont, CA) was introduced as the first commercially available OCT device to map the CE [7]. Other commercially available OCT devices capable of reproducible ETM and their features are summarized in Table 1 (Table 1). There are several reports of custom-built, ultra-high resolution- (UHR-) OCT devices that are used to map the CET but are not commercially available yet [41, 53–56].

### 2.4. Repeatability Studies

Several studies have focused on the repeatability of ETM with VHF-US, SD-OCT, and SS-OCT devices in normal, KCN, post-photorefractive keratotomy (post-PRK), post-laser-assisted in situ keratomileusis (post-LASIK), post-small incision lenticule extraction (post-SMILE), and contact lens (CL) wearer dry eyes (Table 2), utilizing the following objective parameters: (i) “within-subject standard deviation” [43, 63] (SW; the lower the SW, the better the repeatability), (ii) “intraclass correlation coefficient” [48, 52, 56] (ICC; >0.9, good agreement; 0.75–0.9, moderate agreement; <0.75, poor agreement), and (iii) “within-subject coefficient of variation” (WCV; equals SW divided by the average; lower values denote superior repeatability) [52, 67]. The repeatability of a device depends on several factors, including image contrast, penetration rate, axial resolution, tracking ability, and scan density [43]. The repeatability of different devices may decline in the periphery, compared to the central zone [24, 48, 52, 61, 68], and also in post-LRS [43, 52, 61, 63, 67], KCN [43, 62–64, 67], and dry eyes [66], compared to virgin eyes. Table 2 summarizes studies evaluating the repeatability of different devices in different conditions.

#### 2.4.1. Epithelial Thickness Maps of Different OCT Devices

Ge et al. compared RTVue SD-OCT with two other custom-built OCT devices (one ultra-high resolution OCT (UHR-OCT) and one ultra-long scan depth OCT (UL-OCT)) in measuring the central CET and reported good repeatability and similarity of all three devices despite different axial resolutions (5, 3, and 7.5 *μ*m, respectively). They found higher optical resolution of the OCT device to result in better precision and repeatability, by improving the image quality and discrimination of layer boundaries [41].

Feng et al. compared CET measurements in Anterion SS-OCT and Avanti SD-OCT devices in normal, KCN, and post-KRS eyes and found thinner CET measured by Anterion (range: 2.66–4.35 *μ*m), which was more pronounced in 2 to 5 mm diameter. Overall, they found higher repeatability for the Anterion device despite lower axial resolution [43].

#### 2.4.2. Different Algorithms of the Same OCT Device

There are two algorithms of ET evaluation by RTVue SD-OCT: Pachymetry + Cpwr (6.0 mm algorithm) and PachymetryWide (9.0 mm algorithm) scan patterns. In a study by Latifi and Mohammadi [52], these two methods showed good repeatability in CET measurement and a good agreement in the central corneal zone. In the paracentral region, however, there was high variation; the two methods may not be interchangeable.

## 3. Corneal Epithelium in Normal Eyes

Using VHFU technology, Reinstein et al. showed that the average central CET was 53.4 ± 4.6 *μ*m, comparable with SD-OCT [22]. Using SD-OCT, normal CET profiles were described to be thicker in the central 2 mm of the cornea ranging from 53 to 54 *μ*m in the center and decreased with a low gradient of −0.43 *μ*m/mm toward 7 mm midperiphery and a larger gradient of −2.31 *μ*m/mm toward 9 mm periphery [45, 52, 60, 71–73]; VHF-US studies did not find such thinning trends [22].

With rare exceptions [40], almost all studies found thicker CET in the inferior cornea than in the superior cornea of normal eyes [7, 8, 22, 43, 45, 59, 74–82]. This difference may range from 3 to 5.7 *μ*m using VHF-US and 2.2 [80] to 4.4 *μ*m [59] using OCT. This difference is also noted in studies on children of different ethnic groups [45, 65, 83] and was measured by one study as 3.3, 3.5, and 3.6 *μ*m in the paracentral, midperipheral, and peripheral areas, respectively [45]. Some authors attributed this difference to mechanical rubbing of the upper eyelid to superior CE, possibly thinning the superior ET [80, 84, 85].

In the horizontal meridian, the normal CET profile is more controversial. While many authors have found thicker CET in the nasal meridian [22, 50, 65, 75, 76, 78], some have not detected any significant difference between nasal and temporal sides [45, 59, 86]. However, should there be a difference in the horizontal meridian, it has been estimated to be about 1 *μ*m, which is much less significant than the vertical meridian [22].

Using different devices, many studies have explored the possible associations between CET and various factors including aging, gender, and ocular biometric parameters, reviewed below.

### 3.1. Differences between Males and Females

Many studies have found a slightly thicker CET in males [74, 76, 78, 87, 88], while some have not [82, 89, 90]. The reported differences in the central CET between men and women may range from 1.39 to 2.2 *μ*m (*P* < 0.05) [74, 76, 78, 87, 88]. CET is reportedly thicker in male children, according to all studies on children of various ethnic groups [45, 91, 92]. One study found thicker central CET measures in men, compared to women, among both normal and KCN groups [93]; another study reported thinner epithelium in men in peripheral parts of the cornea [90].

### 3.2. Effect of Aging

Although many authors were interested in the effect of aging on the CET profile, this topic remains one of the most debated ones in the literature. While some authors found negligible differences in the majority of zones of the CET profile of adults as a result of aging [22, 74, 76, 85, 94], others found a steady decrease in CET in the older age groups [78, 89]. Two studies described a stable central 2 mm CET and a decrease in paracentral and midperipheral [86, 88] or limbal area [86] due to aging. Loureiro et al. found no significant difference between different age groups of children [45]. However, total CET profiles may show higher variability in older age groups [74, 87, 95], particularly in the superior parts of the cornea [94].

### 3.3. Ocular Biometric Parameters and Refraction

Although CET in almost all sectors was not correlated with the total corneal thickness (TCT) in two studies in adults [82, 96], a study in adults [73] and another in children [83] found a positive correlation between TCT and CET. The study on the pediatric population also reported a negative correlation between CET and white-to-white measurements in Korean children [83].

Kim and coworkers found no difference between CET in low, moderate, and high myopic eyes [88]. Wang et al. also found similar ET profiles in normal and high myopic populations [85]. CET has also been shown not to be associated with axial length (AL) [60, 83, 92] or refractive errors [22, 45, 83, 92], regardless of individuals' age or ethnicity. On the other hand, few studies have reported CET to be negatively correlated with AL and myopia; one possible explanation may be the increased friction between the corneal surface and the eyelid nearing each other with longer axial lengths [73, 78].

No correlation between CET and keratometry has been found in adults [22, 60, 74, 78] or children [83]. Ma and associates found a correlation between the CET and corneal curvature radius among Chinese children in the paracentral and peripheral, but not the central cornea [92]. Hashmani et al. could not find any correlation between different corneal indices, including anterior/posterior surface flat/steep axis, maximum/minimum curvature, corneal astigmatism topography, astigmatism polar values and corneal volume, anterior surface asphericity, and ETM. Posterior surface asphericity was an exception, having a moderate correlation with CET in all areas [60]. On the other hand, Ozalp and Atalay reported a positive correlation between the anterior corneal curvature radius and CET in myopic populations [73].

Sedaghat et al. evaluated the ETM profile in different types of corneal astigmatism and found slightly thicker CET in 180 degrees meridian than 90 degrees in all types of astigmatism, but this difference was statistically significant only in against-the-rule astigmatism group [97]. Yang et al. found a central island of thickening in 9 mm wide ETM of 7.5% of low corneal astigmatism (<2 diopters) and 30% of high corneal astigmatism patients [69]. Yu et al. described significantly thicker CET in the flat axis of the cornea in comparison to the steep axis in refractive laser surgery candidates [98].

### 3.4. Intraocular Pressure

Biomechanical properties of the cornea can affect the accuracy of intraocular pressure (IOP) measurement using applanation methods; some studies have utilized OCT to evaluate the extent to which each corneal layer can contribute to IOP measurement discrepancies [99]. Their results are consistent, pointing out a positive correlation between the measured IOP and the corneal total/stromal thickness, but not the CET [74, 78, 99]. Therefore, despite the weak positive correlation between TCT and CET [78], differences in CET may be too subtle to have a discernible effect on IOP measurement. It may be attributed to the thinner and less rigid (lacking collagen fibrils) nature of the epithelium, compared to the stroma [99].

### 3.5. Diurnal Changes and Patching

Physiological fluctuations in corneal thickness and curvature may occur during the day/night—with a magnitude of around 19 and 22 *μ*m in the central and peripheral cornea, respectively [100]. During sleep time, with reduced uveoscleral outflow and increased IOP, the cornea may be subject to relative hypoxia and increased temperature under closed eyelids and undergo swelling [101, 102]. If present, detecting the pattern and magnitude of such diurnal variations in CET would be important to standardize CET measurement. Existing data on diurnal variations in CET are indecisive. A study using SD-OCT revealed thicker CET measures at 10 AM compared to 4 PM, but this was less than the resolution of the device (i.e., 5 *μ*m) [58]. On the other hand, another study utilizing UHR-OCT (axial resolution ∼ 3 *μ*m) failed to show a statistically significant difference in CET profile at different hours of the day (10 AM, 4 PM, and 6 PM) [75].

### 3.6. Pediatric Age Group

Three studies have evaluated normal ETM profiles in children of different ethnic backgrounds, including 323 Korean [83], 614 Chinese [92], and 60 Portuguese [45] children. These children were 6–17, 8–18, and 7–15 years old, respectively. Most of their findings resembled the adult CET profiles; ETM was thicker in the inferior cornea and in male children, according to all three studies [45, 83, 92]. Only one study found a positive correlation between central CET and aging [92], but the other two did not [45, 83]. Kim et al. suggested that body weight and height were positively correlated with central CET [83]. The other findings of these studies were discussed in previous sections.

### 3.7. Summary

In brief, despite some variation in normal CET values across different devices and populations, the overall average CET is about 53-54 *μ*m in the center, decreasing toward the periphery. Normal eyes may have a thicker CET inferiorly, but nasal and temporal values are less significantly different. In both adult and pediatric age groups, CET may appear slightly thicker in males. Throughout adulthood, CET may decrease with aging, but this is a debated topic. Most studies have not found a significant correlation between CET and TCT, AL, or refractive errors. Lastly, CET may not significantly affect IOP measurement accuracy, and diurnal changes in CET may be indiscernible—if present at all.

## 4. ETM in Corneal Diseases

### 4.1. Keratoconus (KCN)

Several studies aimed to characterize and differentiate ETM changes across different stages of overt KCN as well as subclinical KCN (S-KCN) and *forme fruste* KCN (FFK). Early detection of KCN is essential in preventing accelerating ectasia after keratorefractive surgery (KRS) and in halting ectasia progression by early collagen cross-linking (CXL) [103]. The primary aim of studies in the field was to characterize the changes in ETM patterns in KCN, and the secondary aim was to design algorithms and parameters that would help differentiate normal from KCN corneas (Table 3).

#### 4.1.1. ETM Pattern Changes in KCN

In 2008, Haque et al. were the first to introduce ETM in KCN patients using a TD-OCT machine [40]. In this study, the thinnest CET was described to be in the inferotemporal region in KCN eyes [40]. Numerous studies using SD-OCT and VHF-US described thinning of CE over the cone, which is located inferiorly or inferotemporally, in contrast to the thickening pattern of inferior CET in normal corneas [2, 7, 13, 47, 53, 64, 69, 80, 93, 103, 104, 108, 112, 115–118]. This thinning corresponded to the steepest keratometry [8, 13], maximum axial power [2], maximum mean power [2], highest elevation [8], and thinnest pachymetry point [8, 113]. Thinning of the CET in the inferior-temporal region is reported to be the best single parameter to distinguish between KCN and normal eyes in a study using UHR-OCT [53]. Higher superior to inferior CET in 6 mm and 8 mm sectors has been described as a diagnostic factor for KCN [46]. Nevertheless, in a recent study, the thinnest ET point on ETM with SD-OCT had the poorest correlation with other methods of determining cone location in KCN eyes (i.e., Scheimpflug, scanning-slit, Placido-disk, and OCT pachymetry) [119].

ETM in KCN eyes showed higher variability and standard deviation (SD) [7, 8, 64, 80, 103, 118, 120]. Some authors reported a significant decrease in the thinnest point of the ETM in overt KCN [54, 117], FFK [111], and subclinical KCN eyes compared to normal ones [113]. Moreover, the difference between maximum and minimum (max-min) CET is reported to increase in subclinical KCN [80], FFK [111, 121], overt KCN [7, 47, 93, 95, 111], and posterior KCN [10] compared to the normal subjects. The increase in max-min of more than 13 *μ*m has been reported to be one of the main features of ETM of KCN with 84% sensitivity and 43% specificity [95]. The thinnest to the thickest ETM point ratio was found to be of high diagnostic value in differentiating KCN from normal eyes, with an area under the curve (AUC) of 0.926 [54].

One study introduced root mean square pattern deviation (RMSPD) by comparing individual ETM to mean normal ETM. This index showed maximum AUC (i.e., 1.0) in differentiating normal maps from KCN ones. They also postulated that creating CET pattern deviation maps may facilitate the diagnosis of KCN by enhancing abnormal areas of ETM relative to normal ETM maps [7].

Regarding the central CET, the results are more inconsistent. In some studies, central CET was shown to be thinner in the KCN group [13, 40, 47, 118]; in the others, no difference was found [7, 80]. Kanellopoulos and Asimellis found central CET to be thicker than normal in the milder KCN group and thinner than normal in the more advanced KCN group [64]. Vega-Strada et al. also found thinner central CET as the severity of KCN increased [46].

Using the UHR-OCT map of the central 5 mm of the cornea, Pircher et al. found that the mean thickness of the superior nasal area and maximum CET were the only ET parameters that were thicker in the KCN group [53]. However, Kanellopoulos et al., in two separate reports using VHF-US and SD-OCT, showed that the overall ET is slightly thicker, i.e., 1.1 *μ*m by VHF-US and 0.15 *μ*m by SD-OCT in KCN eyes than normal eyes [64, 120].

With the use of VHF-US technology, Reinstein and colleagues [13, 30] described a typical donut pattern of central thinning and peripheral thickening of CET, which corresponded to the area of the steepest keratometry. The difference between thinning and thickening was shown to be more significant as KCN severity increased. The authors proposed the chafing effect of the eyelids to cause thinning of the CE over the cone. Although the donut pattern of ETM was the typical finding in the papers using VHF-US [13, 30, 104], some studies utilizing SD-OCT technology were unable to find this pattern, although they described thinning of CET over the cone. This controversy was attributed to the limited view of these OCT studies compared to a wider scanning by VHF-US (4–6 mm vs. 10 mm) [7, 54, 115]. Using a custom-built OCT with an 11 mm diameter scan, Pircher et al. found the donut pattern and the moth-like damage pattern of Bowman's layer as the common feature of their KCN cases [54]. Using a 9 mm pachymetry wide scan of SD-OCT, Yang et al. described a similar crater pattern in the ETM in 96% of their KCN cases [111]. Levy et al. evaluated 135 KCN eyes with SD-OCT and mentioned the donut pattern to have 56% sensitivity and 94% specificity in these cases [95].

#### 4.1.2. Discriminating Subclinical and Forme Fruste KCN from Normal

S-KCN defines KCN per topography with absent visual defect (corrected distance visual acuity (CDVA) of 20/20 or better) and normal clinical examinations [7, 112]. On the other hand, some studies have defined the term FFK as topographically normal cornea with normal clinical examinations and CDVA in the contralateral eye of a patient with overt KCN in one eye [8, 112]. Other studies defined FFK as the better eye of asymmetric KCN cases with a normal KISA % (<60%) [2, 106]. Although this is not a common clinical scenario, including only 2–4% of cases, FFK cases have a 50% long-term risk of progressing to overt KCN [7, 25]. As epithelial compensation by acting like a smoothing filter may hinder the diagnosis of FFK, distinguishing FFK from normal corneas is a real challenge for every test designed for KCN diagnosis [8, 25, 105].

Regarding the comparison of FFK eyes with normal ones, some authors found significantly more thinning at the thinnest epithelial point [8, 111] or increased curvature [2] of the cornea. Others found a more inferior thinnest point in FFK eyes [8, 113] and higher ETM standard deviation (SD) and a larger min-max difference [121]. On the other hand, some studies have found no significant remodeling in some or all of FFK eyes [25, 111], and the individual ETM parameters were unsatisfactory in differentiating between normal and FFK eyes [105, 109, 111, 113]. Nevertheless, combining ETM parameters with pachymetry parameters [105, 110, 112] and Bowman's layer indices [55, 114], better discrimination results were found. In a study, combining ETM SD and min-max variables with 11 other OCT pachymetry and Scheimpflug imaging variables yielded the best discrimination power with 100% sensitivity and 100% specificity [109]. Other studies used automated algorithms [25], neural networks, and logistic regression [114]. Some studies have introduced new indices on ETM—or ETM combined with other maps—to differentiate KCN, S-KCN, and FFK eyes from normal eyes. Those indices include, but are not limited to, maximum epithelium ectasia index (EEI-Max) [55], epithelial ectasia index (EEI) [106], epithelial pattern standard deviation (PSD) [106, 107], coincident-thinning index [110], epithelial modulation index (EMI) [112], and epithelial-to-stromal ratio (E/S) [113] (Table 3).

#### 4.1.3. Combined Parameters, Algorithms, and Decision Trees to Differentiate KCN from Normal Eyes

Reinstein et al. showed that ETMs taken by VHF-US technology and analyzed by their automated algorithm successfully detect keratoconus in 5 out of 10 eyes of FFK cases [25]. Silverman et al. evaluated 161 variables of layered corneal pachymetry maps of normal and KCN eyes, including ETM produced by VHF-US technology. After statistical analysis, they chose the 6 variables, including 4 from ETM with the least overlap between the two groups, resulting in a sensitivity of 99% and specificity of 99.5% for the differentiation of the two groups [104]. The same group in another study combined 3 variables of VHF-US, including 2 ETM variables and 4 variables of Scheimpflug imaging, to reach 97.3% specificity and 100% sensitivity. The ETM variables they used were the vertical position of minimum ET and ET gradient at the point of minimum corneal thickness [108].

Elkitkat et al. used a combined Placido-disc topography and SD-OCT device to differentiate between KCN and normal eyes. Although they found ETM parameters to differ between the two groups, these indices were shown to be inferior to many other aberrations, such as pachymetry and elevation indices [47]. Toprak et al. compared ETM produced by a combined SD-OCT and Placido-disc device in 27 FFK and 55 S-KCN with 66 normal eyes. They found the thinnest point to have no difference in the horizontal meridian between the 3 groups but significantly lower in S-KCN than the normal group. The ET data did not differ significantly in central or peripheral CET between the 3 groups except in the inferior and inferior-temporal 5 mm sectors of S-KCN and a superior-nasal sector of the 8 mm zone. In this study, the authors evaluated the E/S ratio in every sector as a novel parameter and found significantly higher E/S in the central cornea in the FFK group. They found a significant superonasal-inferotemporal difference in 5 mm and one sector of 8 mm in S-KCN and FFK, respectively. All of the parameters above failed to discriminate FFK or S-KCN from normal subjects [113].

Xu et al. developed a custom-built ultra-high resolution- (UHR-) OCT with a resolution of 1.1 *μ*m to evaluate the central 4 mm of the cornea. As the device was able to measure Bowman's layer (BL) precisely, they developed several Bowman's, epithelial, and stromal layer indices to evaluate the cornea and distinguish between normal, FFK, and KCN eyes (Table 2). They found excellent discriminative power in all indices when comparing normal to KCN eyes. On the other hand, some indices showed superiority in differentiating FFK from normal eyes, as the changes were more subtle. The maximum epithelium ectasia index (EEI-Max) and the maximum BL ectasia index (BEI-max) had the highest discriminative power. Although BL indices generally had lower diagnostic values than epithelial indices, the authors concluded that adding BL indices might help detect earlier changes in FFK [55]. In another study, combining the same device and Scheimpflug imaging indices and using neural network and logistic regression, the authors compared S-KCN to normal and found epithelial pattern variation to have the best discriminative power. They found the combination of these two systems to offer an AUC of about 0.9 or more in telling S-KCN and normal eyes apart [114]. Using a custom-built OCT with an 11 mm diameter scan, Pircher et al. [54] introduced a color-coded en face BL thickness map (BLTM) in addition to ETM to differentiate KCN from normal. BLTM had a characteristic moth-like change added to the well-known donut pattern of ETM in KCN cases. The thinnest BL point and the ratio of the thinnest to thickest ETM points yielded the highest discriminative power [54].

Hwang et al. compared ETM parameters of 60 normal and 30 FFK eyes by SD-OCT and did not find a statistically significant difference between the two groups except for a higher ETM SD and a larger min-max value. None of the individual ETM parameters showed an acceptable discriminating power between the two groups. Nevertheless, combining the parameters above with 11 other OCT pachymetry and Scheimpflug imaging variables yielded the maximum discriminative power (100% sensitivity and 100% specificity) [109].

Yang and associates designed a 2-step decision tree based on the ETM and pachymetry parameters of the 6 mm SD-OCT (Avanti, Optovue) scans that yielded the highest discriminative power in their previous studies. In the first step, if one of the following parameters were beyond the cutoff, the eye was considered suspicious and evaluated for the second step: (1) minimum pachymetry: 515 *μ*m, (2) difference of minimum and maximum pachymetry: −71 *μ*m, (3) pachymetry superonasal minus inferotemporal: 28 *μ*m, and (4) epithelial SD: 1.88 *μ*m. In the second step, the clinician should inspect the pachymetry and CET maps for coincident thinning. This method resulted in 100% specificity and 97.8% sensitivity in the differentiation of KCN from normal [69, 111]. Utilizing a different SD-OCT device (Zeiss Cirrus 5000 HD), Yücekul and associates used almost the same decision tree with different cutoff points and used superonasal and inferotemporal ET difference instead of ETM SD at the first step, which resulted in 100% sensitivity and 100% specificity in detecting KCN and it had 90.4% sensitivity for subclinical KCN detection [44].

#### 4.1.4. Differentiation of KCN from Contact Lens Warpage (CLW)

CLW can mimic the topographic pattern of KCN because of showing inferior steepening. ETM in KCN and CL wearers was found to have a higher range and variability than normal ETM. The characteristic pattern of ETM in CLW is the inferior thickening of ET over the steep cornea in contrast to inferior thinning in KCN. As a result, focal steepening in CLW is associated with thicker ET [2]. Thickening of inferior CET has been postulated to cause KCN-like changes in topography results of normal corneas in CL or non-CL wearers [122].

Tang et al. found epithelial PSD under 4.1% in 100% of normal eyes in contrast to high PSD (>4.1%) in 100% of KCN and 81.8% of CLW eyes. They introduced the “epithelial ectasia index (EEI)” and “warpage index (WI)” and found high EEI and low WI in KCN and FFK eyes not wearing CL. CLW eyes had high WI in contrast to KCN and FFK eyes. The contact lens wearer FFK and KCN eyes had both indices positive [106]. Later, the same team introduced the epithelial modulation index (EMI), which was based on the covariance of the ET and mean curvature deviation. They combined epithelial PSD and EMI to differentiate between FFK, S-KCN, CLW, and KCN cases. This combination successfully classified all CLW cases and all KCN, S-KCN, and FFK eyes, except 3 FFK cases with high PSD, misclassified by normal range EMI. Mean EMI was statistically similar and negative for CLW and normal eyes; it was higher—and positive—in overt KCN, S-KCN, and FFK, in descending order [112].

#### 4.1.5. KCN vs. PMD

A study compared the ETM of 10 PMD with 59 KCN cases and compared them with a 9 mm wide pachymetry SD-OCT system after matching maximum keratometry. They found acceptable device repeatability in both groups, with better repeatability results in the center that decreased toward the periphery. The comparison of ETM between the two groups showed significantly lower ET in the 7–9 mm sector of PMD eyes and lower ET in the inferior 2–5 mm sector of KCN eyes. The best single parameter to differentiate the two conditions was ET in the 7–9 mm sector (sensitivity: 80%, specificity: 73%, cutoff: 53.7 *μ*m); an inferior 7–9 mm ET of ≤54 *μ*m and inferotemporal ET of ≥55 *μ*m strongly indicated a diagnosis of PMD [68].

#### 4.1.6. Mild vs. Severe Overt KCN

In an SD-OCT study, KCN eyes with worse CDVA had thinner central CE, thicker 8 mm superior, and thinner 8 mm inferior. In other words, the higher the KCN grading, the thinner the CET and the higher the superior-inferior in 6 mm and 8 mm [46].

In another report, patients were divided into milder or more severe and were compared with normal subjects. The authors found higher central CET in the milder cases than in normal and more severe groups and lower central CET in those with more severe KCN. They also found higher ET variability among more severe cases [64]. In another recent study, the authors found the central CET and the ETM to be thinner and more variable, respectively, in higher KCN severities—except for scarring KCN, which resulted in a thicker ET [123].

#### 4.1.7. Progressive vs. Nonprogressive KCN

Ouanezar et al. divided mild and moderate KCN cases into progressive or nonprogressive groups and compared SD-OCT and scanning-slit device indices between these groups. They defined progression as a 1.0 diopter increase in steepest keratometry over 6 months. Among all epithelial indices, the central CET variation and the thinnest CET variation indices were significantly narrower in the progressive KCN group. Nevertheless, these two indices showed lower performance than the thinnest corneal thickness to detect KCN progression, per ROC curve analyses [124]. Serrao et al. longitudinally evaluated progression over one year and compared progressive and nonprogressive KCN cases. They found paracentral inferior ET to be significantly thinner in progressive KCN [93].

#### 4.1.8. Changes after CXL without Other Procedures

Reinstein et al. were the first to propose the ETM measured by VHF-US technology as a tool to monitor progression, which may occur after CXL. They noticed a slight decrease in both thinning and peripheral thickening in CET of their patient with post-LASIK ectasia in two-year follow-up, which they supposed to be a sign of a halt of progression [28]. Rocha et al. compared preoperative and 3-month postoperative ETM measured by SD-OCT of 17 KCN and 14 post-refractive ectasia eyes; they found significantly thinner CET at several locations 1 to 2.5 mm all around the corneal apex and lower ETM SD (3 to 6 *μ*m) in the 3-month postoperative visit. They attributed the changes above to a more regular cornea due to successful CXL [103]. Atia et al. described similar thinning of CET in several sectors of 3 mm and 6 mm zones of ETM as an indicator of more effective epithelium-off CXL; on the other hand, thinning occurred only in the inferior sectors of iontophoresis-assisted transepithelial CXL at 6-month follow-up [125]. Lautert et al. described a significantly decreased min-max value in ETM measured by SD-OCT at 6-month follow-up of their 93 KCN patients. The min-max parameter decrease strongly correlated with maximum keratometry readings on Scheimpflug imaging [126]. While a study using accelerated CXL by epithelium-off method (30 mW/cm2 for 4 minutes) found thinner CET in multiple nasal and inferior sectors and lower ETM SD in 12-month follow-up [127], others using transepithelial accelerated ETM could not find such changes in the same [128] or longer [129] follow-up periods.

#### 4.1.9. Changes after CXL with Other Procedures

The combination of CXL with PRK (i.e., Athens protocol) for myopic eyes caused an increase in minimum CET and inferior CET while it decreased CET variability; these changes were deemed to be a result of a more regular cornea [130–132]. Although two studies found significant central thickening of ETM following Athens protocol [5, 130], one study could not find such alterations [131]. In one study comparing the 12-month postoperative ETM of combined trans-PRK (T-PRK) and 90 seconds of CXL to stand-alone T-PRK, less epithelial hyperplasia and myopic regression were associated with the combined technique—it was attributed to CXL [5]. Similarly, in a study comparing 6-month postoperative ETM of combined LASIK and 80 seconds of CXL to stand-alone LASIK, the authors found significantly less epithelial hyperplasia in midperipheral CET in the combined group in high myopic cases of more than 7 diopters [133].

### 4.2. Corneal Dystrophies

A study compared 45 epithelial basement membrane dystrophy (EBMD) eyes to normal and dry eye disease (DED) cases. This study found thicker ET values in the EBMD group except for superior and minimum ET. As a result of ROC curve analysis, irregularity of epithelium had the best discrimination with a cutoff of above 3.1 *μ*m in EBMD group [134]. In another study, the authors compared the ETM pattern of 55 EBMD to other ocular surface disorders and found inferior thickening (sensitivity of 55% and specificity of 92%) and increased central ET of more than 56 *μ*m (sensitivity of 53% and specificity of 81%) [95]. There are case reports regarding increased ET in Schnyder corneal dystrophy, Reis–Bucklers dystrophy, and Meesmann corneal dystrophy [33, 135–137]. A weak correlation between changes in the ETM and contrast sensitivity was found in one study, but there was no correlation with CDVA [138].

### 4.3. Limbal Stem Cell Deficiency

Limbal stem cells (LSCs) play a vital role in the maintenance of homeostasis of the ocular surface. They are primarily located in limbal crypts between the palisades of Vogt (POV) in the superior and inferior limbus. The epithelial thickness of POV is higher in the superior and inferior quadrants compared to the nasal and temporal limbus. Moreover, an age-dependent change is observed in POV epithelial thickness; it increases up to the age of 40 and then declines in the seventh decade of life [139]. The loss or dysfunction of limbal stem cells characterizes limbal stem cell deficiency (LSCD), leading to persistent corneal epithelial defects, corneal conjunctivalization and vascularization, corneal opacity, and decreased vision.

Corneal epithelial thickness and limbal epithelial thickness decrease in patients with LSCD. Moreover, the decrease in CET correlates with the severity score of LSCD [140]. Epithelial mapping of eyes with LSCD using SD-OCT has shown increased max-min ET and ET SD compared to normal eyes [141]. Levy et al. found that LSCD is associated with a spoke-wheel pattern on the epithelial thickness map, max-min ET above 14 *μ*m, and an ET SD of more than 5 *μ*m [95].

### 4.4. Miscellaneous Corneal Disorders

Evidence is still scarce regarding ETM applications in several corneal disorders; below is a brief review of the few works that explored OCT-generated ETM utilities in those clinical contexts.

In a report of 5 patients with recurrent epithelial erosion, hot spots (areas of increased ET) on ETM were used to guide anterior stromal puncture successfully [142].

Lu and Palioura used SD-OCT (ETM and pachymetry) to differentiate the cause of persistent epithelial defect in herpetic keratitis; they selected patients for steroid treatment based on increased stromal thickness and irregular ETM with good treatment outcomes [143].

An SD-OCT study measured ETM changes in 16 DSAEK patients over 6 months; dramatic decreases in the ETM SD and difference of minimum and maximum thickness were found—resulting in a more regular ETM. Compared to the early postoperative period, the mean CET in the center decreased by about 9.7 *μ*m in this study [144].

In a report, the ETM of normal individuals was compared to normal fellow eyes of patients with unilateral keratopathy. The authors found significantly thinner central epithelium and thicker central stroma in these seemingly normal fellow eyes and introduced higher central E/S as a possible indicator of subtle endothelial dysfunction in these eyes [145].

In a report of 10 patients with carcinoma in situ, with histopathologic confirmation, a maximum CET above 60 *μ*m (sensitivity of 91% and specificity of 60%) and an ETM SD above 5 *μ*m (sensitivity of 100% and specificity of 58%) were reported to be the main topographic features [95]. In another report, a thickening pattern over the affected area was reported as the main feature of squamous hyperplasia [146].

Levy et al. described nasal thickening patterns with 100% sensitivity and 86% specificity in 10 cases of pterygium. Nasal ET of more than 56 *μ*m yielded 80% sensitivity and 71% specificity for detecting pterygium in these cases [95].

## 5. Refractive Surgery

### 5.1. To Proceed or Not to Proceed

Reinstein et al. were the first to use ETM to make the vital decision to proceed to keratorefractive surgery (KRS). They screened their patients by routine topography techniques and, after excluding overt KCN cases, selected 84% of borderline cases with normal ETM on VHF-US. They matched this borderline group with a completely normal topography group and followed them for one year after performing LASIK. Both groups showed similar safety profiles; ectasia occurred in neither [34].

In a recent paper, Asroui et al. highlighted the potentially pivotal role ETM may play in modern KRS decision making. They documented the changes in the decision making for KRS planning after combining ETM with other imaging methods, e.g., Scheimpflug imaging. They reported a 16% change in the KRS candidacy by two masked examiners, including 10% inclusion and 6% exclusion of patients. Furthermore, ETM effectively changed the ranking of the most favorable KRS to the least in 25% of cases, with an 11% gain of eligibility for LASIK and a 6% loss [147].

### 5.2. Planning the Laser Device

Reinstein et al. reported successfully utilizing stromal maps (i.e., the difference in surface elevation of corneal topography and ETM) to treat a patient with irregular astigmatism following complicated LASIK. They planned to perform LASIK based on the map under a thicker flap than the first operation [31].

To address the CET changes in central areas in patients with residual refractive error following LRS, Zhou et al. used ETM-assisted topography-guided T-PRK to treat 70 eyes with residual refractive errors following LRS. This treatment includes two parts: (i) the lamellar or PTK part and (ii) the refractive part to address residual myopia, astigmatism, and aberration of higher order. The difference in CET at the thickest part and the ablation depth was used to set the ablation depth of the correction. The surgery resulted in a safe, effective improvement of the residual refractive errors [148].

In a contralateral eye study, our team compared the effect of programming the laser device with the actual CET profile in the center and periphery for T-PRK in virgin right eyes compared to the default protocol of the device, which considered CET 55 *μ*m in the center and 65 *μ*m in the periphery in the left eye. We did not find a significant difference in wasted tissue, but slightly hyperopic results and more contracted optical zones were noted [72].

### 5.3. Changes in Refractive Results after Refractive Surgery Based on Different ETM Measurements

Cleary and associates used regression analysis to determine the association of central and peripheral CET, measured by SD-OCT, and the difference between the intended and the actual spherical equivalent of laser correction. They found a range of refractive effects between +1.07 and −1.91 D from epithelial thickness variation. More precisely, if the central CET is thinner than the periphery, the phototherapeutic keratectomy (PTK) resembles myopic LRS, and vice versa—a thicker CET at the center resembles hyperopic LRS [149].

Jun et al. evaluated the refractive outcomes of two groups of eyes with thicker (>60 *μ*m) and thinner (<50 *μ*m) CET, which underwent wavefront-guided ablation T-PRK using the same excimer device with the default 55 *μ*m CET settings. No differences in safety and efficacy or induced higher order aberration were found; all eyes achieved ±0.5 D of the intended value, but the difference in the postoperative sphere and spherical equivalent was significant. They found a slight myopic shift (−0.05 D) in the thicker CET group and a slight hyperopic shift (+0.05 D) in the thinner CET group [150].

### 5.4. ETM in Planning PTK Procedure

There are several reports of using VHF-US ETM as a guide to discerning the amount of epithelial masking and underlying stromal irregularity to treat the irregularities of the cornea better. Reinstein and Archer introduced ETM-assisted transepithelial PTK (TE-PTK) in patients with compensatory ETM changes due to highly irregular stroma. The cases had a history of multiple refractive procedures [151], previous RK [18], corneal irregularities [3, 152], or truncated/complicated LASIK flaps [21, 152]. This procedure aims to break through the thinnest points of CE to expose the highest points of the stromal irregularities while avoiding full-thickness epithelium removal. They defined the CET of 51 to 60 *μ*m in highly aberrated corneas as the therapeutic window and found 55 *μ*m as the suitable initial ablation depth [21]. Furthermore, by subtracting the ETM profile from the anterior corneal topography, they aimed to address the irregularities of the underlying stroma and avoid suboptimal treatment by topography or wavefront-guided ablation alone [3, 4, 18]. In some cases, they used a series of 6 s PTK after flooding the corneal surface with BSS in a technique called wet PTK [12].

The drawback of the TE-PTK in the highly irregular cornea included unpredictable refractive outcomes, with 23% hyperopic or 17% myopic shifts and 41% of the patients with ±0.50 diopters of emmetropia [12]. The additional refractive surgery can be performed at the same or next sitting to refine the correction and gain the best visual results, and this second step could consist of TE-PTK, standard refractive, wavefront, or topography-guided ablation [12, 18, 151].

### 5.5. Changes in ETM following Surface Ablation

Chen et al. were the first to use SD-OCT ETM to evaluate epithelial and stromal thickness profile changes after PRK [153]. Almost all studies have shown increased epithelial thickness after PRK. Increased epithelial thickness was reported in patients who had undergone PRK more than 20 years ago [154]. Chen et al. [153], Hou et al. [155], Sedaghat et al. [63], and Weng et al. [156] documented this increase up to 1, 3, and 6 months after PRK, respectively, with ongoing recovery. Latifi and Mohammadi [52] proposed an 18-month model to describe the process: (i) epithelial removal causes an initial decrease in CET 1 month after myopic PRK, (ii) the epithelial thickness gradually begins to increase until reaching stability in variable time points, and (iii) the change in the mean CET stabilizes at month 6 in the midperipheral and peripheral zones and month 12 in the central zone—in the paracentral zone, it continued to increase even after 18 months after surgery [71].

Chen et al.'s study [153] revealed a more pronounced thickening of the CET pericentrally compared to the central area. Hou et al. [155] represented it as a negative meniscus-like, lenticular pattern with less central thickening, increasing progressively toward the midperiphery. Sedaghat et al. [63] also showed a lenticular shape that mirrors the 6 mm ablation zone; the thickness of the midperipheral and peripheral zones reached preoperative levels, whereas the thickness in the central 5 mm area was significantly thicker after operation. Latifi and Mohammadi [52] found the thickest epithelium in the paracentral zone at all time points, followed by the central, midperipheral, and peripheral zones.

Weng et al. reported a correlation between slower epithelial recovery and higher baseline spherical equivalent refraction [156]. Chen et al. [153] found a trend toward greater epithelial thickening with a larger amount of programmed SE correction, smaller treatment zone, and thinner preoperative epithelium. Hou et al. [155] found a significant positive relationship between epithelial thickening and changes in *Q* value (measure of corneal asphericity) 6 months postoperatively. Sedaghat et al. [63] found a correlation between the changes in epithelial thickness and spherical equivalent in the paracentral and peripheral zones from before to 6 months after PRK. Increased epithelial thickness changes in the paracentral zone were associated with increased spherical equivalent, while changes in the peripheral zone were accompanied by decreased spherical equivalent. Latifi et al. [52, 71] confirmed previous findings of an association between epithelial thickening and the degree of myopic correction, but no association between the ablated axis of astigmatism and change in respective mean meridional thickness was seen.

Laser platforms and surgical techniques can also alter this epithelial thickness change. Shetty et al. [157] showed higher degrees of epithelial thickness distortion in the Streamlight group than in the SmartSurfACE one. Lu et al. [158] investigated the effect of mitomycin-C (MMC) in epithelial remodeling. MMC significantly reduced corneal haze after PRK and altered epithelial thickness in the first month, but there was no difference in epithelial thickness after 3 months, and MMC had no effect on epithelial remodeling.

### 5.6. Changes after LASIK

#### 5.6.1. Epithelial Thickness Pattern after Myopic LASIK

Multiple studies have investigated epithelial thickness profile alterations induced by myopic LASIK. Most of them found postoperative epithelial thickening with a lenticular pattern across the central 6 mm, as Reinstein and colleagues reported for the first time [17, 34, 159–162]. The epithelium thickens across the central 6 mm—with maximum thickening centrally and progressively less thickening centrifugally—stabilized between 3 and 12 months, and no change in epithelial thickness occurred after 3 months [17]. Tang et al. also emphasized that the epithelial thickening peaked in the central 4 mm diameter and tapered off toward the periphery; the maximum epithelial thickening occurred at an annular area about 3-4 mm in diameter, not exactly at the center [160]. They postulated that the actual LASIK ablation pattern might be designed to compensate for the laser-induced spherical aberration, which means that the actual ablation at the paracentral area would be deeper than Munnerlyn's algorithm used in the simulation, which did not account for spherical aberrations.

In contrast to previous findings, Kanellopolous and Asimellis reported the epithelial thickening as a “negative meniscus-like lenticular” pattern, with more significant thickening at the midperiphery than at the center. They attributed the differences to the specifics of the refractive ablation and instrumentation, the measurement technique (noncontact AS-OCT vs. saline immersion ultrasound), and the number of meridians employed to create thickness maps (8 meridians vs. 4) [163].

Most studies reported a positive correlation between central epithelial thickening and spherical equivalent refraction treated [34, 159, 160, 163, 164]. Tang et al. predicted the extent of epithelial remodeling by a mathematical model correlated with the amount of LASIK correction [160]. Saleh et al. stated that greater improvement in UDVA was associated with increased central epithelial thickening [164]. However, García-Basterra et al. measured the epithelium with MS-39 and found no correlations between the degree of myopia and epithelial thickening [162], as described by Reinstein et al.; they ascribed this to the tear film interfering with accurate epithelial measurements by OCT devices. In addition, corneal epithelial changes in their study were less than 6 microns in all sectors (except temporal); thus, the study might have been underpowered to clarify this point [34, 159, 160, 163, 164].

Reinstein et al. emphasized that although the epithelium thickened progressively with increasing central ablation depth, paradoxically, the gradient of epithelial thickening from center to periphery was steeper for low myopia than for moderate and high myopia. Consequently, the myopic refractive shift due to epithelial profile changes was more significant in low myopic ablations. They found that the rate of thickening (mean thickening per diopter treated) decreased with increasing myopia [34]. Kanellopolous and Vingopoulos investigated the effect of pregnancy on corneal characteristics after LASIK. They found no change in refractive error, corneal stability, and total corneal and epithelial thickness in women after LASIK [165].

#### 5.6.2. Epithelial Changes and Myopic Regression

Reinstein et al. reported that the lenticular epithelial changes contributed to the observed myopic shift after myopic LASIK during the first 3 months [17]. Cho et al. also proposed that the difference between CET at the central and midperipheral zones might play a role in the final refractive error regardless of the time point of the postoperative examination. However, they emphasized that there was only 0.28 D of myopic undercorrection per each 18 mm greater thickness of the central epithelium. Thus, the influence of CET on refractive error is still debated [161].

To better understand the effect of corneal epithelial thickening on myopic regression after LASIK, Ryu et al. investigated the reduction in corneal epithelial thickness during medical treatment for myopic regression following femtosecond- (FS-) LASIK. They found that corneal epithelial thickness decreased proportionally with the magnitude of improvement of myopic regression during treatment with steroid and antiglaucoma drugs in post-LASIK eyes with myopic regression. They even showed that the subgroup with the thickest epithelium (≥62 *μ*m) showed a higher success rate and greater changes in refraction and vision [166].

#### 5.6.3. Epithelial Thickness Patterns after Hyperopic LASIK

Reinstein et al. investigated epithelial changes after hyperopic LASIK correction and found that the average epithelial thickness profile showed an epithelial donut pattern characterized by localized central thinning within the 4 mm diameter zone surrounded by an annulus of thick epithelium. The amount of epithelial thickening per diopter after hyperopic LASIK was more noticeable than that reported after myopic LASIK and similar to that seen in advanced keratoconus [23].

### 5.7. Changes after SMILE

Several studies evaluated the CET profile following SMILE operation for myopia and astigmatism and found central thickening of CE [20, 49, 98, 167–170]. Ganesh et al. evaluated the CET profile after the correction of myopic astigmatism by SMILE and observed central and superior thickening after 3 months of follow-up, which was correlated with the degree of myopia. The thickening was 6.83% for low, 9.26% for moderate, and 12.7% for high myopia [167]. On the other hand, Ye et al. found the largest amount of thickening in the paracentral zone (2–5 mm), followed by central (2 mm) and midperipheral (5–7 mm) zones. The thickening would reportedly begin on the first day postoperatively, increase until 3 months, and stabilize with no significant change until 6 months [98, 169]. Yu et al. detected an increase in the CET of the peripheral annulus (7–9 mm) [98], unlike Ye et al., who described a CET decrease in the same region [169]. Yu et al. found a positive correlation between residual stigmatism after the surgery and the difference of steep and flat corneal meridians in preoperative midperipheral CET profile [98]. Luft et al. described the majority of thickening in the first 6 weeks, stabilizing during the first 3 months following SMILE surgery [49, 50]. The level of refractive correction and the combination of patient's age and spherical equivalent of the extracted lenticule predicted epithelial hyperplasia in this study [49]. Regression in high myopic eyes has been attributed to epithelial hyperplasia in 4 eyes with myopia higher than 8 diopters [167].

### 5.8. Studies Comparing SMILE vs. LASIK

Kanellopoulos et al. evaluated the ETM of 21 patients after myopic LRS in a contralateral eye study, performing FS-LASIK for one eye and SMILE for the other. He described similar ETM thickening magnitude, mostly in the midperipheral cornea, that stabilized in the third postoperative month and during the two-year follow-up period. LASIK eyes demonstrated a greater thickening in the paracentral area during the first three months, and SMILE eyes showed lower variation and a more homogenous CET profile during the first 12 months [171].

Ryu et al. evaluated postoperative ETM of two groups of FS-LASIK and SMILE surgeries for myopia and found a significant increase in the central, paracentral, and peripheral zones of both groups with a linear association with preoperative refractive errors. They found a larger increase in central and paracentral CET in the LASIK group and a more pronounced increase in the midperipheral zone in the SMILE group—it was attributed to the larger treatment zone in SMILE despite optical zone matching. The topographic variability was higher in the LASIK group in this study [62].

### 5.9. Changes after RK

Thickening of the central epithelium has been reported to occur up to 26 years after radial keratotomy (RK), and these changes seem to be a long-term response to the curvature change, in contrast to what occurs after surface ablation and LASIK [15, 16, 18].

### 5.10. Changes after Corneal Inlays

Evaluation of ETM in myopic eyes following intracorneal ring segment (ICRS) implantation by VHF-US technology has shown relative thinning of ET over the segment and thickening of ET inside the ring area [35]. A recent study evaluated ETM changes in 68 KCN eyes after the insertion of ICRS of different arc lengths over 6 months. They found ET reduction over the ICRS and relative thickening adjacent to it, which was greater on the internal site, often exceeding 65 *μ*m. This remodeling began immediately after insertion and no further smoothing of epithelium occurred over the following months. Although they also found a thickening over the cone which was attributed to more regular underlying stroma, there was no correlation between different topographic and refractive parameters and remodeling of the epithelium [172].

Two studies assessed the ETM profile changes after lenticule addition keratoplasty procedures for KCN and high hyperopia. Evaluation of ETM by combined Placido-disc and OCT device 6 months after stromal addition of negative meniscus-shaped lenticule in 15 KCN eyes revealed an increase in the central CET and outer annular area, as well as a reduction in midperipheral CET in areas with increased anterior corneal curvature [173]. In the other study, adding lenticules to treat 10 moderate-to-high hyperopic eyes resulted in donut-shaped changes in the epithelium characterized by thinner central and paracentral 5 mm zones compared to the outer 7 mm zone. As a result, the thinnest CET decreased, and the thickest CET increased significantly [96].

## 6. Contact Lens Use

### 6.1. Short-Term Effects

Eight hours of wearing daily disposable soft contact lenses (SCL) of different brands may not result in significant ETM alterations [174]. On the other hand, using mini-scleral contact lenses (MSCL) during the first 8 hours have shown to cause trivial initial edema in the first 30 minutes and significant thinning in the subsequent 8 hours in central 4 mm CET in both higher (more than 335 *μ*m) and lower (less than 335 *μ*m) corneal clearance groups. Although no significant difference in CET parameters was noted among either group, there was a trend toward less thinning in the higher clearance group [175].

Wearing silicone hydrogel SCL for 7 consecutive days and 6 nights in 3 diopters myopic eyes caused no significant difference in ETM profile, while in the 3 diopters hyperopic group, it showed significant CET thinning in the center, in most of the paracentral zone and inferotemporal sector of midperipheral zone, but significant thickening in some sectors of peripheral CET [176].

### 6.2. Long-Term Effects

Long-term usage of SCL (an average of 4 years in one study and 60.58 ± 40.98 months in another) can cause significant thinning of CET profile in all central, paracentral, and midperipheral sectors [177, 178]. One study showed this thinning to occur in some sectors of ETM of patients using SCL for less than a year, compared to all sectors of >1 year users. This thinning was present in the sixth month of follow-up after SMILE surgery without adverse effects on the results of the surgery [81]. One study found higher variability (min-max) in long-term SCL users [178], compared to normal eyes, while another could not find this change [177]. Although removal of the SCL after two years of usage had a significant thickening effect on the thinned CET profile due to CL use, the CET was significantly lower than that of the control non-SCL user group [179]. Long-term use of hard contact lenses (HCL) exerted similar thinning effects on the central and paracentral zones; no significant change in midperipheral zones or variability of the cornea occurred [178]. The CET profile was not different between <10 year and >10 year RGP users [40].

### 6.3. Orthokeratology (OK)

Alterations in the ETM profile associated with OK lenses in treating low-to-moderate myopia (<5 diopters) and <1.5 diopters of astigmatism have been investigated. In the central 2 mm zone, almost all studies have reported decreased central CET after the use of OK lenses for myopic eyes [6, 29, 91, 148, 180–185]. Regarding the paracentral region (2–5 mm), results are less consistent; different findings from different studies suggest no significant change [180], significant decreases in thickness in temporal [6, 181, 184], nasal [181], and inferior sectors [184], nonsignificant increase in CET along the vertical meridian [181], and significant thinning in the paracentral zone with more prominence in temporal and inferior sectors [91]. Concerning the midperipheral region (5-6 mm), findings from a handful of studies include no significant change [180] and superior and inferior [6, 184] thickening [183] with prominence in the nasal sector than temporal [91, 185].

Increased min-max has been reported by some studies [180, 184], particularly in OK lens users for more than two weeks [184]. Although some studies reported a significant increase in the corneal stromal thickness after OK lenses [91, 181], there was no significant change in stromal posterior surface radii of curvature [181], and the refractive changes after OK were caused mainly by changes in CET and anterior stromal curvature and not correlated with stromal thickness alteration [91, 181].

One study compared the ETM changes between vertical and horizontal meridians after OK use in corneas with “with-the-rule” astigmatism and found that paracentral zone thinning was more prominent in the horizontal meridian (flat) than vertical (steep) meridian. In addition, midperipheral thickening was less significant in the horizontal meridian than in the vertical meridian [148].

In one study comparing the changes of ETM as a result of OK in hyperopic eyes with less than 1 diopter to myopic eyes, central thickening and midperipheral thinning occurred, but these changes were not statistically significant, perhaps due to the small number of cases [183].

Some studies evaluated the effect of the duration of OK lens use on the ETM profile. Interestingly, ETM changes of OK became significant even after 15 minutes of lens wearing in the center and after 30 minutes in the midperiphery [183]. On the other hand, removing the OK lenses reversed the aforementioned ETM changes in central and paracentral zones after 3 hours [185]. Studies have reported that central CET thinning lasted as long as one month [180] and 3 months [6] using OK lenses, but the greatest decrease (thinnest CET) was seen after one week [6, 180]. Significant thinning in the paracentral region was found as early as day 1, limited to one sector (temporal), which extended to more sectors after one week (all in the temporal half of the cornea) and was stable afterwards—for up to 3 months [6]. Thickening in the midperipheral region continued to progress until one month and then became stable [6, 180]. Another study reported the same finding: thinning of the central CET did not continue after 2 weeks of OK lens wearing, and midperipheral CET thickening was more pronounced in those who wore OK lens for more than 2 weeks compared to less than 2 weeks [184]. Comparing OK lens-wearing groups of 2–4 weeks, 5–12 weeks, and >12 weeks, Kim et al. showed a continued trend of thinning in the center and the paracentral area, although it was not significant [91].

One study evaluated the possible correlation of corneal pigmented arc seen in OK lens wearers with 9 mm ETM and found a significant positive correlation between the severity of pigmentation and the inferior sector of midperipheral CET and inferior and inferotemporal sectors of the peripheral zone. It was also shown that the pigmented arc severity was negatively correlated with central CET and positively correlated with OK-lens duration [186].

A study evaluating myopia progression prediction with CET changes after OK lens wear found more pronounced CET changes in the paracentral and midperipheral cornea after wearing OK lenses to be associated with less myopia progression in the future [187].

In contrast to findings in myopic eyes, CET changes after OK lenses in hyperopic eyes (<1 diopter) included central thickening and midperipheral thinning—although not statistically significant [183].

## 7. ETM in Other Ocular Conditions

### 7.1. Eyelid Abnormalities

Comparison of ETM in 30 eyes with allergic conjunctivitis with their normal counterparts using OCT showed a significant decrease in average CET of paracentral (2–5 mm) and midperipheral (5–7 mm) annuli, with a negative correlation with eye rubbing frequency and allergic sign severity. The allergic group also had a higher ETM SD [188].

Comparison of ETM of 13 eyes with mild congenital myogenic ptosis (<2 mm) to 13 normal eyes showed significantly thinner ET and higher ETM SD, yet both groups had similar total ET. The authors attributed these changes to the thinning of the superior ET in all sectors in the ptosis group compared to inferior sectors [189]. Focal thinning in the central CE due to a large central chalazion of the upper eyelid has been reported; it resolved completely after successful treatment of the lesion [190].

### 7.2. Dry Eye

CE is more irregular than normal in patients with DED, which correlates with symptoms of DED, corneal fluorescein staining, and Schirmer test results [191]. Several reports indicated a superior epithelial thinning pattern in DED [80, 95, 192]. Cui et al. assessed the features of ETM in DED patients with different severity grades. They found decreased superior CET in DED compared to normal subjects, which was more significant in higher grades of DED. They attributed this finding to more frequent blinking in patients with DED, leading to thinning of superior epithelium by mechanical friction. Moreover, they postulated that increased thickness of tear film in the superior cornea might induce CE remodeling [192].

Similarly, Edorh et al. reported that CET measured by OCT is significantly thinner compared to normal subjects, regardless of location (i.e. central, inferior, and superior zones). However, the difference between superior and inferior quadrants in DED did not differ from the normal population. Furthermore, the thickness of peripheral corneal epithelium correlated well with Schirmer I score and tear film break-up time (TBUT). Interestingly, the difference between inferior and superior peripheral zones could be used to assess the severity of DED. Patients with grade 1 DED showed hyperplasia of superior CET, while more severe stages (grades 2 and 3) were associated with progressive thinning of superior CET compared to inferior zone [193]. In contrast, Kanellopoulos and Asimellis found that overall CET (including central epithelial thickness, average epithelial thickness, minimum epithelial thickness, and maximum epithelial thickness) was greater in patients with DED [77]. These contradictory results may have roots in the different OCT mapping techniques and that Kanellopoulos et al. did not assess the severity of DED and, thus, might have included patients with low-grade DED.

Rattan and Anwar reported an increased inferior CET and CET variability in DED. This finding may be due to poor tear film function, leading to epithelial proliferation and increased cellular layers in the inferior cornea [80]. Liang et al. evaluated ocular surface epithelium in DED. They reported a decreased limbal epithelial thickness in patients with DED, while CET at the bulbar conjunctiva had increased. Moreover, they did not find any change in corneal epithelial thickness (CET) of the DED group compared to normal subjects [194]. CET and CET variability can serve as objective measures for monitoring the treatment of DED. CET and TVT decreased significantly 6 months after treatment with 0.05% topical cyclosporine in patients with post-LASIK DED [171].

### 7.3. Topical Medications

Evaluation of one-week administration of topical loteprednol on the various corneal parameters of seasonal allergic conjunctivitis revealed no statistically significant effect of the drug on the central CET [195]. Several studies have evaluated the effects of antiglaucoma medications on the ETM, most of which found a generalized thinning pattern of CET [196–199]. Although some studies reported CET thinning as an early event after initiation of the drug [196], not related to the type and duration of the therapy [196, 199] or number of medications and administered instillation [199], others found multiple factors associated with CET thinning [197, 198]. The number of medications reportedly affected all central, paracentral, and peripheral zones, and the duration of instillation affected the central and peripheral zones' CET [197]. In a more specific study, *β* blockers, prostaglandins, and the number of daily benzalkonium chloride-containing instillation affected the CET the most [198]. Cennamo et al. used the combination of ETM and the number of microvilli on scanning electron microscopy to evaluate the effect of glaucoma medications on the eye and found a thicker CET profile in the patients with fewer microvilli numbers [200].

### 7.4. Cataract Surgery

Although phacoemulsification is a safe surgical procedure, the corneal surface is exposed to several intraoperative (e.g., mechanical intraoperative trauma, multiple irrigations, etc.) and postoperative (e.g., multiple doses of eye drops containing preservatives) factors, entailing epithelial remodeling [201]. Some studies with different designs have evaluated ETM changes after cataract surgery [133, 201–203], almost all reporting an increase in the central and midperipheral CET with a gradual decrease to baseline levels within a month [133, 201–203]. However, their reported back-to-baseline time points varied; some studies report a return to preoperative levels within 3–7 days [202, 203], while some others report longer durations ranging from 5–15 days [201, 204] to 4 weeks [133]. In addition, CET reduction to baseline levels can take longer in people with diabetes [202].

## 8. Systemic Conditions

### 8.1. Systemic Diseases

The effects of systemic factors on corneal epithelial thickness have not been extensively studied. We reviewed the current evidence in the literature to demonstrate the impact of some systemic conditions on corneal epithelial thickness.

In a study, the authors found significantly thicker central, paracentral, and midperipheral CET and a similar increase in stromal thickness in patients with diabetes compared to normal group [205]. Another study demonstrated reduced corneal thickness in adolescents with thalassemia major; high serum transferrin levels and liver iron concentrations were associated with reduced corneal thickness [206]. Another study found decreased epithelial thickness in patients with Graves' disease regardless of orbitopathy presence. It has been suggested that subclinical chronic inflammation in people with Graves' disease could play a role in ocular surface status [207].

Systemic lupus erythematosus can significantly reduce corneal central and epithelial thickness. One study demonstrated that SLE patients with or without clinical dry eye had thinner corneal epithelium than healthy controls [208]. Another study found no significant difference in epithelial thickness between patients with DED ± ocular graft-versus-host disease [209].

Psoriasis has not shown an impact on ocular surface epithelial thickness. However, it may increase corneal stromal thickness [210]. CET was observed to increase in epidermolysis bullosa patients; the authors recommended using AS-OCT for corneal assessment to evaluate the effectiveness of new treatments in clinical trials [211].

### 8.2. Systemic Medications and Toxins

AS-OCT has been recommended to monitor ocular surface adverse effects in patients with relapsed or refractory multiple myeloma (RRMM) receiving belantamab mafodotin (belamaf). In a study by Matsumiya et al., increased corneal epithelial thickness following belamaf treatment was demonstrated using AS-OCT measurements. Another study by Mencuci et al. demonstrated a transient increase in epithelial thickness in RRMM patients on belamaf followed by a subsequent diffuse decline [204, 212].

Systemic isotretinoin treatment may increase corneal epithelial thickness and decrease stromal thickness—referred to as the “remodeling of corneal layers” by Ozyol et al. [213].

In a study by Munsamy et al., acute use of e-cigarettes did not result in a statistically significant alteration in corneal epithelial thickness measured immediately after use. The authors suggested that more research is required to assess the impact of more frequent exposure on corneal epithelial thickness [214].

## 9. Conclusion

Our knowledge of ETM in normal eyes and eyes with corneal and systemic diseases is undoubtedly one of the most expanding fields in the anterior segment subspecialty, and evaluating CET is gaining a critical place in the diagnosis and management of corneal diseases.

Some compensatory rules of the CE, mostly described by Dr. Reinstein and associates, are always worth mentioning when we describe the changes in CET to provide a smoother ocular surface and compensate for the irregular underlying stroma; these compensatory rules may be classified as follows: (i) thickening in areas of flattening or removed tissue [14, 71, 159], (ii) thinning over the areas of steepening, elevation, or added tissue [23, 35, 172], (iii) the amount of CE remodeling depends on the rate of curvature alterations [18], and (iv) when the refraction is stable in irregular astigmatism, the CE has reached the maximum compensatory status [31].

Understanding normal values and variation patterns in CET is essential for interpreting the information provided by ETM. The clinical applications of this information are quite extensive; ETM data can improve the diagnosis and management of several corneal disorders, including KCN, dystrophies, and LSCD. For instance, analysis of ETM data and/or pattern alterations may help (i) distinguish KCN from contact lens warpage and/or PMD, (ii) characterize different KCN stages, (iii) differentiate cases with mild vs. severe overt and progressive vs. nonprogressive KCN, and (iv) evaluate the efficacy of CXL and combined procedures.

Furthermore, the safety and efficacy of refractive surgeries can be improved by incorporating ETM data by (i) better identifying borderline cases for KRS, (ii) planning laser devices and optimizing clinical processes in complicated cases, and (iii) monitoring postoperative CET changes. The utilization of ETM has enabled researchers to investigate and compare changes in CET profiles after different refractive surgery procedures. It also can aid in understanding the effect of corneal epithelial thickening on refractive regression and guide clinical decision making.

Another topic of research interest has been the pattern, magnitude, and reversibility of changes in CET profile based on the type and duration of contact lens wear. Other active research areas include the impact of other ocular conditions and interventions on CET profile, such as allergic conjunctivitis, mild congenital myogenic ptosis, chalazion, DED, and topical glaucoma medications.

Although how systemic factors may influence the corneal epithelium and the clinical indications for such effects remain less understood, some conditions, like diabetes, Graves' disease, and systemic lupus erythematosus, have been associated with CET changes. Existing data point out some systemic medications, e.g., belantamab mafodotin and isotretinoin, to alter the corneal epithelium profile, with probable implications for monitoring ocular adverse effects. As our knowledge about CET is new and rapidly expanding, the exact mechanisms of such responses to systemic diseases or medications are yet to be characterized and understood.

## 10. Search Strategy

We used the PubMed database for our search. We performed the search in the time interval between the introduction of the first ETM in the literature (1993) and July 2022 (the time of the last search) using several keywords, including Corneal epithelial thickness: 1459 results, cornea epithelial thickness: 1306 results, Corneal epithelial map: 241 results, corneal epithelial mapping: 136 results, corneal epithelial thickness mapping: 72 results, and corneal epithelial thickness profile: 126 results. We evaluated the titles and abstracts of all studies and included all human studies (case reports, case series, and original studies) with available English abstracts. Full-text evaluation of 401 relevant studies was then performed. In this study, we aimed to review the reproducible ETM studies utilizing SD-OCT or VHF-US. We excluded OCT studies that manually measured the corneal epithelial thickness (CET) (e.g., by digital calipers) or the CE (e.g., by confocal scanning or handheld pachymeters). Among full texts, 59 studies were excluded by one researcher (MAA) because of irrelevant or unreproducible data using manual techniques than automatic ET measurements in the studies or insufficient data about epithelial thickness maps or preproof studies. The studies were categorized into 39 folders regarding the topics and were then reviewed.

## Figures and Tables

**Table 1:** Optical coherence tomography devices currently used in corneal epithelial thickness mapping.

Device name	Technology	Axial resolution (*μ*m)	Wavelength (nm)	Scan diameter (mm)	Speed (A-scan/second)/acquisition time (s)
Artemis 2 (Arc scan) [30]	VHF-US	1	Ultrasound	10	NM/120 to 180
Anterion (Heidelberg Engineering) [43]	SS-OCT	10	1300	7	50,000 <1
Avanti/RTVue (Optovue, Inc) [43]	SD-OCT	5	840	6–9	Avanti: 70,000 RTVue: 26,000 0.58
Cirrus 5000 (Carl Zeiss Meditec) [44, 45]	SD-OCT	5	840	9	68,000 NM
MS39 (CSO) [46, 47]	Combined Placido-based topographer and SD-OCT	3.6	845	8	30,000 NM
REVO NX (Optopol Technology) [48]	SD-OCT	5	830	8	110,000 0.17
RS 3000 (Nidek Co) [49, 50]	SD-OCT	4	NM	8	53,000 NM

NM: not mentioned, OCT: optical coherence tomography, SD: spectral domain, and VHF-US: very high-frequency ultrasound.

**Table 2:** Studies on the repeatability of different devices in eyes with different conditions.

Author/year	Device type/brand	No. of subjects	Significant findings
*Studies on normal eyes only*			
Shen et al. 2013 [56]	RTVue (SD-OCT) and CB-OCT (UHR-OCT)	NL: 18	ICC: CB-OCT: 0.82–0.97 RTVue: 0.78–0.92 (Higher repeatability toward the center)
Kanellopoulos and Asimellis 2013 [57]	RTVue (SD-OCT)	NL: 373	SW: 0.88 ± 0.71 *μ*m in the center
Wasielica-Poslednik et al. 2015 [58]	RTVue (SD-OCT)	NL: 23	ICC: 0.780–0.952
Beer et al. 2018 [59]	CB PS-OCT	NL: 20	WCV ≤3% (Very good repeatability)
Hashmani et al. 2022 [60]	RTVue (SD-OCT)	NL: 220	ICC: Inner circle: 0.703–0.812 Middle circle: 0.712–0.917 Outer circle: 0.796–0.930 (Good-to-excellent reproducibility)
Sikorski 2022 [48]	REVO NX, Optopol (SD-OCT)	NL: 137	ICC: 0.64–0.95 (Higher repeatability toward the center)

*Studies on post-LRS eyes ± normal eyes*			
Reinstein et al. 2010 [23]	Artemis VHF-US	Post-LASIK: 10	SW: 0.58 *μ*m at the corneal vertex 0.43–1.36 *μ*m at the central 6 mm
Ge et al. 2013 [41]	RTVue (SD-OCT), Visante (TD-OCT), and 2 CB-OCT devices (UHR-OCT and UL-OCT)	NL: 20 Post-LASIK: 18	ICC: >0.84 SW: <2.2 *μ*m in NL <4.8 *μ*m in post-LASIK (Higher *optical* resolution ∼ better repeatability)
Ma et al. 2013 [61]	RTVue (SD-OCT)	NL: 35 Post-LASIK: 45	ICC: ≥0.917 in normal ≥0.891 in post-LASIK
Ryu et al. 2017 [62]	RTVue (SD-OCT)	Post-FS-LASIK: 62 Post-SMILE: 113	SW: 0.8 *μ*m at center 1.2–1.5 *μ*m at midperiphery (Higher repeatability toward the center)
Sedaghat et al. 2018 [63]	RTVue (SD-OCT)	Pre and post-PRK: 52	SW: 1.73 *μ*m pre-PRK 4.50 *μ*m 6 months post-PRK
Latifi and Mohammadi 2020 [52]	RTVue (SD-OCT) (6 mm vs. 9 mm scan)	Myopic: 95 Post-PRK: 117	WCV: In 6 mm scan: <3.08% (myopic) and <4.80% (post-PRK) In 9 mm scan: <5.14% (myopic) and <5.18% (post-PRK)

*Studies on eyes with ocular surface disorders ± post-LRS or normal eyes*			
Li et al. 2012 [7]	RTVue (SD-OCT)	NL: 75 KCN: 35	SW: 0.7 *μ*m in NL 1.0 *μ*m in KCN (at central 2 mm)
Kanellopoulos and Asimellis 2014 [64]	RTVue (SD-OCT)	NL: 160 KCN: 160	SW: 0.89 *μ*m in NL 1.78 *μ*m in KCN (at central 2 mm)
Ma et al. 2018 [65]	RTVue-XR (SD-OCT)	NL: 12 CL user: 12 DED: 11 Post-PRK/LASIK: 12 KCN: 14	SW: ≤2.6 *μ*m in all groups 0.7–1.5 *μ*m in NL 1.0–2.6 *μ*m in CL users 0.9–2.0 *μ*m in DED 0.7–2.5 *μ*m in post-PRK/LASIK 1.1–2.3 *μ*m in KCN (highest in inferior peripheral zones, except for KCN eyes)
Sella et al. 2018 [66]	RTVue (SD-OCT)	NL: 12 CL user: 12 DED: 12 Post-LRS: 12 KCN: 12	SW: 0.9 *μ*m in NL 1.2 *μ*m in eye with corneal conditions (at central 2 mm) Similar repeatability in all groups, except DED eyes with lower repeatability (2.0 and 2.4 *μ*m in superior and inferior 2–5 mm ring for DED vs. 0.8–1.3 and 0.8–1.1 *μ*m for other groups); an inverse correlation with DED severity was suggested
Vega-Estrada et al. 2019 [46]	MS-39	NL: 60 KCN: 107	SW: 1.24 *μ*m in NL 2.03 *μ*m in KCN (at central 3 mm)
Lu et al. 2019 [67]	RTVue-XR (SD-OCT)	NL (myopic): 75 Post-PRK: 68 Post-SMILE: 61 Post-LASIK: 75 Mild KCN: 20 Advanced KCN: 53	WCV: 1.7–3.5% in NL (myopic) 2.3–6.3% in post-LRS 2.5–6.2% in mild KCN 3.5–8.0% in advanced KCN (More variable in post-LRS and KCN eyes)
Mohr et al. 2020 [68]	RTVue-XR (SD-OCT)	KCN: 59 PMD: 10	ICC: 0.827–0.986 in KCN 0.753–0.998 in PMD (Reduced repeatability toward the periphery)
Li et al. 2021 [69]	REVO NX, Optopol SD-OCT	KCN ± CXL: 212	ICC: >0.86 in KCN/CXL− >0.83 in KCN/CXL+
Schiano-Lomoriello et al. 2022 [70]	MS 39 SD-OCT	KCN: 44	WCV < 5% (for central CET)
Feng et al. 2022 [43]	Anterion SS-OCT and Avanti SD-OCT	NL: 90 KCN: 122 Post-LRS: 46	SW: By Anterion: 0.88 (NL), 1.08 (post-LRS), 1.26 (KCN) *μ*m By Avanti: 1.12 (NL), 1.62 (post-LRS), 1.52 (KCN) *μ*m (In 0–7 mm area) (Anterion was superior)

CB: custom-built, CL: contact lens, CXL: collagen cross-linking, DED: dry eye disease, FS: femtosecond, ICC: intraclass correlation coefficient, KCN: keratoconus, LASIK: laser-assisted in situ keratomileusis, LRS: laser refractive surgery, NL: normal, OCT: optical coherence tomography, PMD: pellucid marginal degeneration, post: postoperative, pre: preoperative, PRK: photorefractive keratectomy, PS-OCT: polarization sensitive OCT, SD: spectral domain, SMILE: small incision lenticule extraction, SS: swept source, SW: within-subject standard error, UHR-OCT: ultra-high resolution OCT, UL-OCT: ultra-long OCT, VHF-US: very high-frequency ultrasound, and WCV: within-subject coefficient of variation.

**Table 3:** Studies evaluating specific indices or methods in the diagnosis of FFK, S-KCN, and KCN vs. normal eyes.

Author, year	Device	Significant indices or algorithms	Comparison eyes	Results
Li et al. 2012 [7]	SD-OCT 6 mm D	RMSV: root mean square of variation RMSPD: root mean square pattern deviation	76 NL 35 KCN	All indices were positive in KCN RMSPD has the greatest AUC

Silverman et al. 2014 [104]	VHF-US 10 mm	6 variables, including 4 ETM variables analyzed by linear discriminant analysis (LDA) Neural network (NN) analysis	130 NL 74 KCN	LDA: 100% AUC NN: 100% AUC

Temstet et al. 2015 [8]	SD-OCT 6 mm	Location and CET of thinnest	42 NL 36 FFK 32 severe KCN	Inferior thinnest CET in 91.3% Thinnest central CET was thinner in FFK compared to NL

Catalan et al. 2016 [105]	SD-OCT 6 mm	Combination of 7 ETM and other pachymetry variables	104 NL 22 FFK 22 KCN	No good discrimination of individual ETM variables A combination of ETM and pachymetry had good discrimination power

Tang et al. 2016 [106]	SD-OCT 6 mm D	Epi-PSD (pattern standard deviation) Ant. ectasia index Warpage index	22 NL 31 KCN 11 CLW 8 FFK	Epi-PSD: NL <4.1% vs. KCN, CLW, FFK >4.1% Ant. ectasia index: KCN > FFK > CLW > NL Warpage index: positive in CLW vs. negative in KCN, FFK

Li et al. 2016 [107]	SD-OCT 6 mm D	Epi-PSD Corneal PSD Stromal PSD	83 NL 50 S-KCN 1 FFK	All 3 parameters were successful Epi-PSD had the greatest AUC

Xu et al. 2016 [55]	UHR-OCT	EEI: epithelium ectasia index BEI: Bowman's layer ectasia index EEI-max: maximum epithelium ectasia index BEI-max: maximum BL ectasia index	81 NL 37 KCN 32 FFK	EEI-max and BEI-max had the highest power of discrimination

Silverman et al. 2017 [108]	VHF-US and Scheimpflug device	3 variables of VHF-US including 2 ETM variables and 4 variables of Scheimpflug imaging	111 NL 30 KCN	97.3% specificity 100% sensitivity

Hwang et al. 2018 [109]	SD-OCT 6 mm D and Scheimpflug imaging	Two ETM parameters (ETM SD and greater min-max) and 11 other OCT pachymetry and Scheimpflug indices	60 NL 30 FFK	None of the individual ETM parameters showed a good discrimination power Combination of parameters: 100% sensitivity and 100% specificity

Vega-Strada et al. 2019 [46]	SD-OCT+ Placido disc	Thinner 3 mm central Greater S-I (8 mm)ratio Greater S-I (6 mm)ratio	60 NL 107 KCN	Combined 3 parameters: AUC: 0.92

Pircher et al. 2019 [54]	UHR-OCT	BLTM Minimum BL thickness R1ET (thinnest to thickest ET)	20 NL 47 KCN	Moth-like change in BLTM Min BLT: AUC: 0.983 R1ET: AUC: 0.926

Pavlatos et al. 2020 [110]	SD-OCT	Coincident thinning index	82 NL 133 KCN, S-KCN, FFK	CTN: KCN > S-KCN > FFK > NL

Yang et al. 2020 [111]	SD-OCT	2-step decision tree	54 NL 19 FFK 11 S-KCN 91 KCN	NL: 100% specificity KCN: 97.8% sensitivity S-KCN: 100% sensitivity FFK: 73.3% sensitivity

Pavlatos et al. 2021 [112]	SD-OCT 6 mm	Epi-MI (modulation index) Epi-PSD	32 NL 20 CLW 89 KCN 16 S-KCN 26 FFK	Epi-PSD: NL<4.1%, KCN, CLW, FFK> 4.1% Epi-MI: NL=CLW << FFK < S-KCN < KCN

Toprak et al. 2021 [113]	SD-OCT+ Placido disc	E/S (epithelium to stroma) ratio	66 NL 27 FFK 55 S-KCN	ETM parameters failed to show a good discrimination power between FFK or S-KCN and normal

Yücekul et al. 2022 [44]	SD-OCT	2-step decision tree	172 NL 20 S-KCN 172 KCN	100% sensitivity, 100% specificity in KCN 90.4% sensitivity in S-KCN

Shi et al. 2022 [114]	UHR-OCT and Scheimpflug imaging	Epithelial pattern variation (EPV) Analysis: neural network Logistic regression	50 NL 33 S-KCN 38 KCN	EPV: best discrimination power Combination OCT and Scheimpflug: AUC about 0.9

AUC: area under the curve, BL: Bowman's layer, BLTM: Bowman's layer thickness map, CET: corneal epithelial thickness, CLW: contact lens wearer, ETM: epithelial thickness mapping, FFK: forme fruste KCN, KCN: keratoconus, KCN: keratoconus, NL: normal eye, RMSPD: root mean square pattern deviation, RMSV: root mean square of variation, SD: standard deviation, SD-OCT: spectral-domain OCT, S-KCN: subclinical KCN, UHR-OCT: ultra-high resolution OCT, and VHF-US: very high-frequency ultrasound.

## Data Availability

No underlying data were collected or produced in this study.
